# Pheochromocytoma masquerading as fulminant myocarditis with marked interleukin-6 elevation: a case report

**DOI:** 10.1093/ehjcr/ytag798

**Published:** 2026-10-26

**Authors:** Yuka Tanizaki, Tokutada Sato, Sayuri Nii, Hiroshi Suzuki, Yoshitaka Iso

**Affiliations:** Division of Cardiology, Department of Internal Medicine, Showa Medical University Fujigaoka Hospital, 1-30 Fujigaoka, Yokohama, Kanagawa 227-8501, Japan; Department of Intensive Care Medicine, Showa Medical University Fujigaoka Hospital, 1-30 Fujigaoka, Yokohama, Kanagawa 227-8501, Japan; Department of Intensive Care Medicine, Showa Medical University Fujigaoka Hospital, 1-30 Fujigaoka, Yokohama, Kanagawa 227-8501, Japan; Division of Diabetes, Metabolism, and Endocrinology, Department of Internal Medicine, Showa Medical University Fujigaoka Hospital, 1-30 Fujigaoka, Yokohama, Kanagawa 227-8501, Japan; Division of Cardiology, Department of Internal Medicine, Showa Medical University Fujigaoka Hospital, 1-30 Fujigaoka, Yokohama, Kanagawa 227-8501, Japan; Division of Cardiology, Department of Internal Medicine, Showa Medical University Fujigaoka Hospital, 1-30 Fujigaoka, Yokohama, Kanagawa 227-8501, Japan

**Keywords:** Case report, Fulminant myocarditis, Cardiogenic shock, Pheochromocytoma, Catecholamine-induced cardiomyopathy, Interleukin-6

## Abstract

**Background:**

Pheochromocytoma is a rare cause of cardiogenic shock with diverse cardiovascular manifestations. Although catecholamine-induced cardiomyopathy is well recognized, presentations closely resembling fulminant myocarditis are uncommon and diagnostically challenging. The role of cytokine-mediated inflammatory activation in such presentations is not well defined.

**Case summary:**

A previously healthy woman in her 30s presented with fever, ventricular arrhythmias, and severe left ventricular dysfunction, rapidly progressing to cardiogenic shock requiring venoarterial extracorporeal membrane oxygenation and Impella support. Fulminant myocarditis was initially suspected based on clinical, electrocardiographic, and biomarker findings, and immunomodulatory therapy was initiated. Although transient improvement was observed, endomyocardial biopsy showed minimal inflammatory infiltration. Subsequent computed tomography revealed a left adrenal mass, and biochemical testing confirmed markedly elevated catecholamines. Notably, interleukin-6 (IL-6) was also markedly elevated relative to C-reactive protein. Despite initiation of α-blockade, the patient developed recurrent haemodynamic collapse, necessitating emergency adrenalectomy. Pathological examination confirmed a necrotic pheochromocytoma, but the patient died of refractory multiorgan failure.

**Discussion:**

This case illustrates how pheochromocytoma can closely mimic fulminant myocarditis, with a risk of diagnostic anchoring. The coexistence of marked IL-6 elevation and catecholamine excess raises the possibility of concomitant inflammatory activation, although a causal role of IL-6 cannot be established. Recognition of this pattern is important to prompt reconsideration of endocrine aetiologies in myocarditis-like cardiogenic shock.

Learning points

- Pheochromocytoma can present as fulminant myocarditis–like cardiogenic shock even without sustained hypertension, with a risk of diagnostic anchoring.
- Marked interleukin-6 elevation may accompany this presentation, although its source and mechanistic significance remain uncertain; discordant findings during the clinical course should prompt diagnostic reassessment.

## Introduction

Pheochromocytoma is a catecholamine-secreting tumour with diverse clinical manifestations and is a rare cause of acute heart failure and cardiogenic shock. Although catecholamine-induced cardiomyopathy is well recognized,^1^ presentations that closely resemble fulminant myocarditis are uncommon and diagnostically challenging. In most reported cases,^2,3^ myocardial dysfunction is primarily attributed to catecholamine toxicity, and the role of inflammatory activation has been less clearly defined. We report a case of pheochromocytoma presenting as myocarditis-like cardiogenic shock with marked cytokine elevation.

## Summary figure

**Figure:**
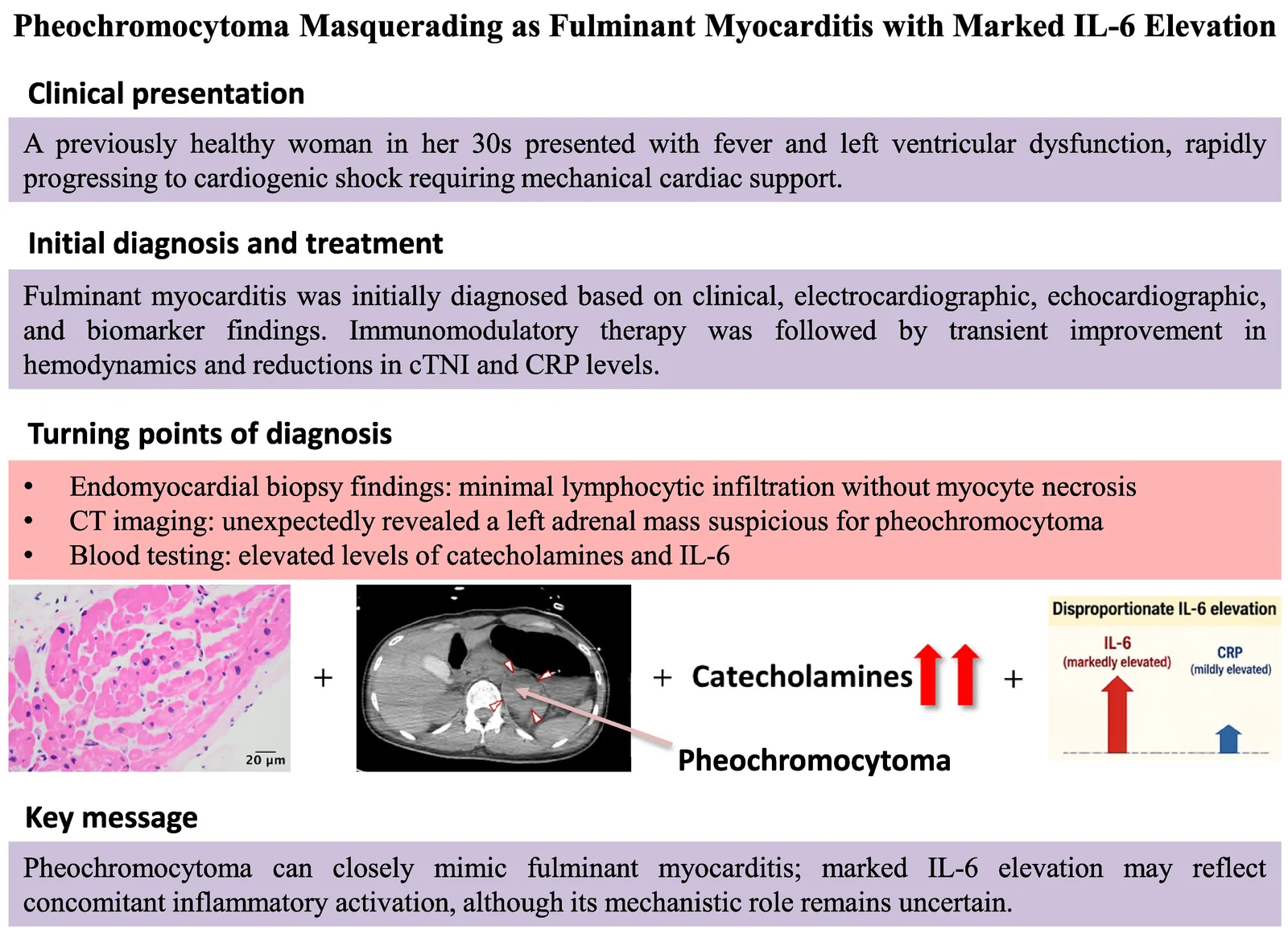


## Case presentation

A previously healthy woman in her 30s was referred to our hospital with a fever of 38°C, profound fatigue, nausea, dyspnoea, and frequent premature ventricular contractions. She had a history of gestational hypertension that resolved postpartum without ongoing antihypertensive therapy. Eight years earlier, computed tomography (CT) performed at our hospital for suspected urinary tract infection had shown no adrenal enlargement or mass.

On arrival, she appeared acutely ill with a heart rate of 122 b.p.m., blood pressure of 146/103 mmHg, and peripheral oxygen saturation of 96% on room air. Electrocardiography demonstrated sinus tachycardia with non-specific ST-segment elevation (*Figure 1A*). Peripheral blood testing revealed elevated cardiac troponin I (cTNI, 8086 pg/mL), brain natriuretic peptide (63.2 pg/mL), and C-reactive protein (9.35 mg/dL). Transthoracic echocardiography demonstrated severe global left ventricular systolic dysfunction with an ejection fraction of ∼10% (see Supplementary material online, *Movie S1*). Shortly after presentation, she developed sustained ventricular tachycardia and ventricular fibrillation, followed by profound cardiogenic shock requiring cardiopulmonary resuscitation in the emergency department. Mechanical circulatory support was rapidly instituted with venoarterial extracorporeal membrane oxygenation (VA-ECMO) and an Impella CP device. Emergency coronary angiography revealed no obstructive coronary artery disease. Fulminant myocarditis was considered the most likely diagnosis, and endomyocardial biopsy was performed. Electrocardiography after intensive care unit admission showed marked ST–T abnormalities (*Figure 1B*), further supporting this working diagnosis.

**Figure 1:**
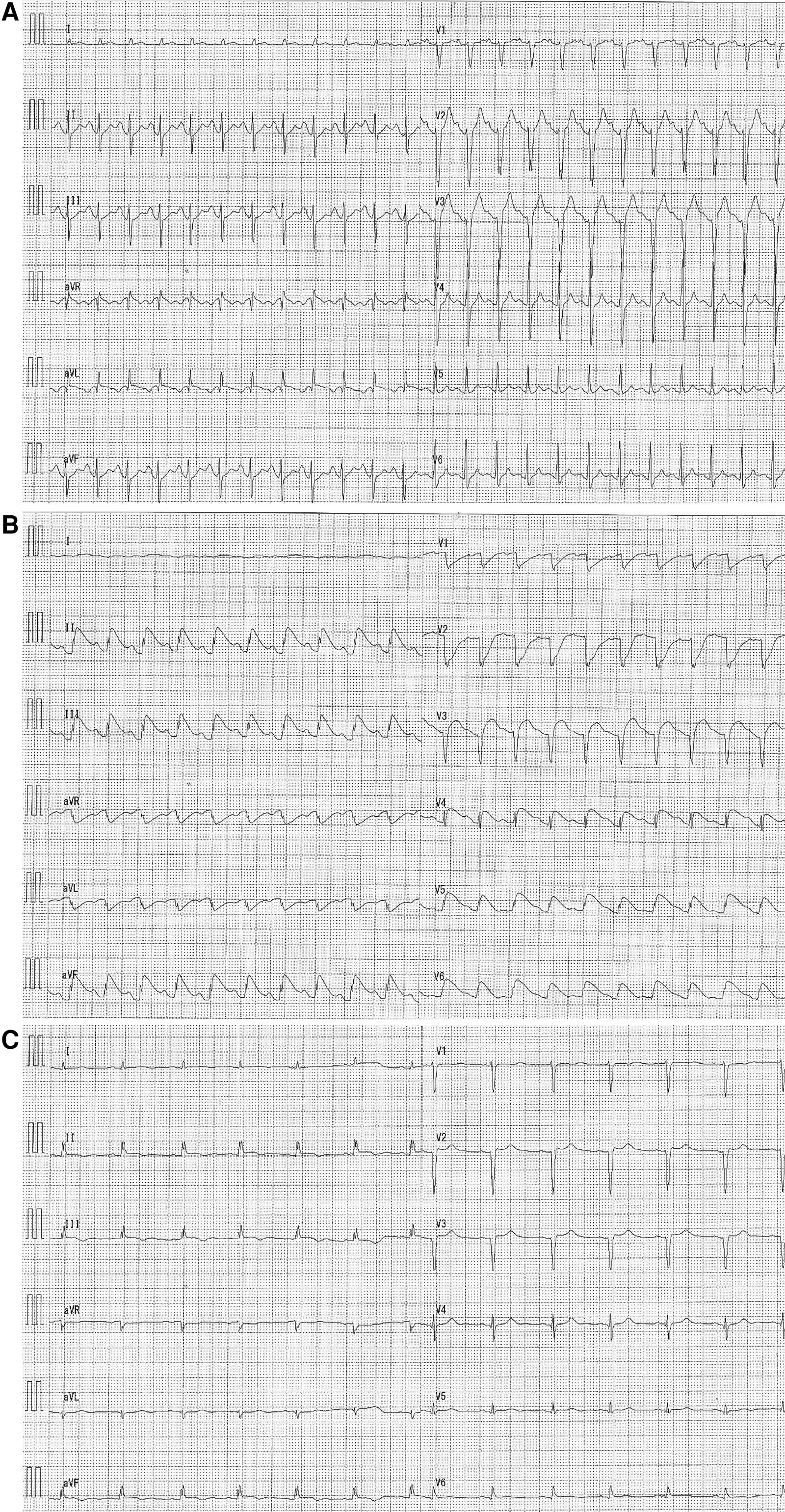
Electrocardiography. Electrocardiograms obtained (*A*) at presentation, (*B*) on admission to the intensive care unit, and (*C*) on Day 6 before venoarterial extracorporeal membrane oxygenation withdrawal.

The time-course changes in cTNI and C-reactive protein levels and points of clinical events are shown in *Figure 2*. Representative serial haemodynamic parameters, mechanical circulatory support settings, vasoactive drug requirements, and laboratory data are provided in Supplementary material online, *Table S1*. High-dose corticosteroid pulse therapy (methylprednisolone 1000 mg/day for 3 days) was initiated immediately after admission under ongoing mechanical circulatory support, followed by intravenous immunoglobulin therapy for 3 consecutive days. Biomarker levels decreased transiently, accompanied by improvement in systemic perfusion. ST–T abnormalities partially improved by Day 6 (*Figure 1C*), and VA-ECMO was successfully withdrawn while Impella 5.5 support was continued (see Supplementary material online, *Movie S2*). At this stage, the clinical course appeared consistent with steroid-responsive fulminant myocarditis. However, endomyocardial biopsy findings demonstrated minimal lymphocytic infiltration without myocyte necrosis (*Figure 3*), and viral serologic testing did not support acute viral infection.

**Figure 2:**
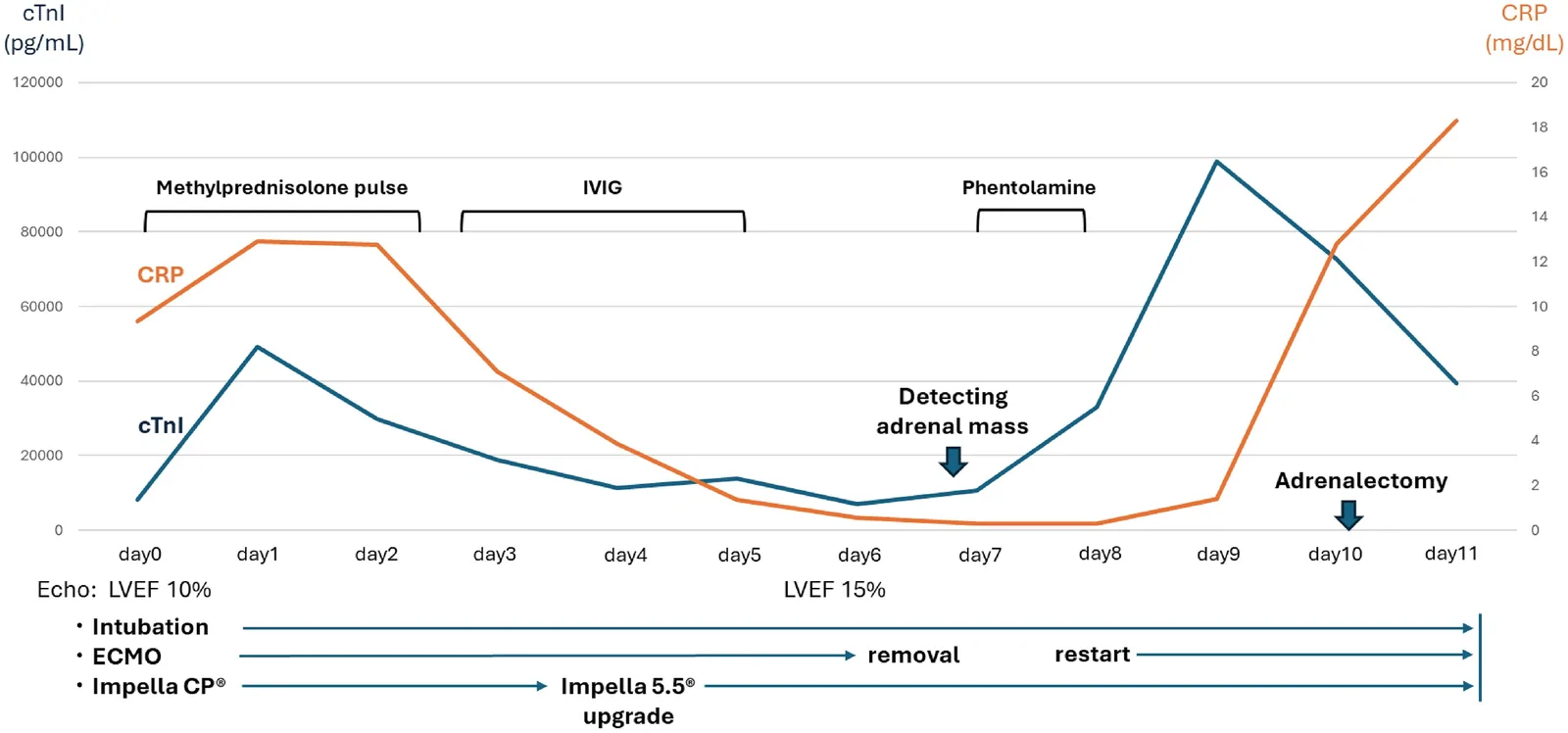
Clinical timeline and biomarker changes. Clinical course and trends in cardiac troponin I and C-reactive protein during hospitalization in a patient with fulminant myocarditis–like cardiogenic shock requiring intubation and mechanical circulatory support with venoarterial extracorporeal membrane oxygenation and Impella devices. LVEF, left ventricular ejection fraction.

**Figure 3:**
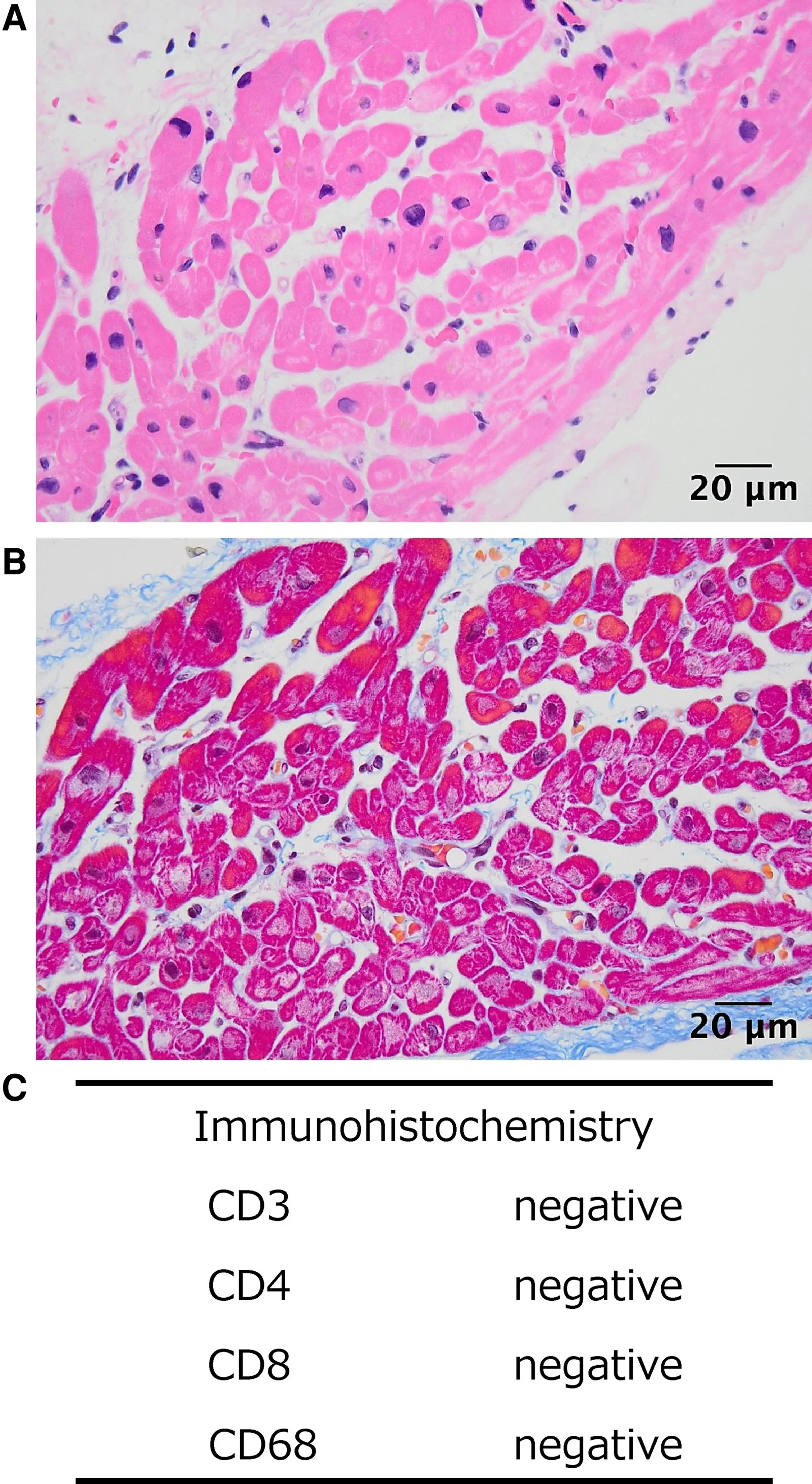
Endomyocardial biopsy findings. (*A*) Haematoxylin–eosin staining. (*B*) Masson trichrome staining. (*C*) Summary of immunohistochemical analysis for CD antigens in lymphocytes and macrophages.

During recovery from the initial shock state, the patient developed progressive abdominal distension consistent with paralytic ileus. Computed tomography imaging from the chest to pelvis was obtained after ECMO decannulation to evaluate for potential procedural complications and abdominal pathology. Imaging unexpectedly revealed a left adrenal mass suspicious for pheochromocytoma (*Figure 4A*). Subsequent biochemical testing, performed while exogenous noradrenaline and dobutamine were not being administered, demonstrated markedly elevated plasma concentrations of catecholamines and free metanephrines (*Table 1*; Supplementary material online, *Figure S1*).

**Figure 4:**
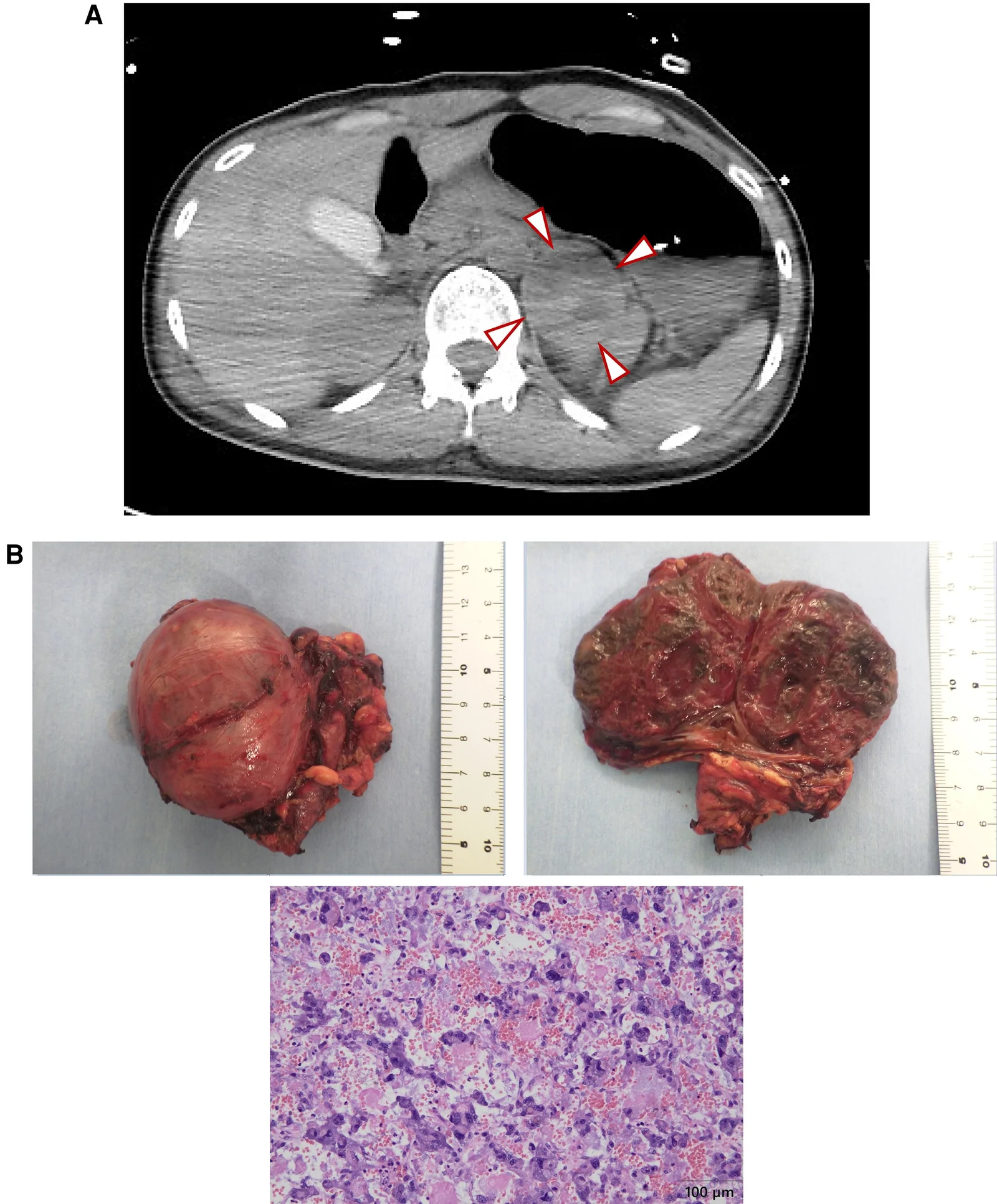
Adrenal mass on computed tomography and resected tumour. (*A*) Axial computed tomography showing a left adrenal mass detected after venoarterial extracorporeal membrane oxygenation removal. The triangular markers (△) indicate the mass suspicious for a pheochromocytoma. (*B*) Gross and histopathological findings of the resected adrenal tumour. The microscopic image shows pheochromocytoma (haematoxylin and eosin staining; scale bar, 100 μm).

**Table 1:** Blood levels of catecholamines, free metanephrines, and IL-6

	Results	Reference range
Epinephrine, pg/mL	38 000	≤100
Norepinephrine, pg/mL	83 320	100–500
Dopamine, pg/mL	2240	≤30
Free metanephrine, pg/mL	3536	≤130
Free normetanephrine, pg/mL	6258	≤506
IL-6, pg/mL	245	≤7.0

Despite the absence of sustained hypertension, pheochromocytoma was suspected, and intravenous phentolamine was initiated at 2 mg/h on Day 7 and subsequently adjusted according to haemodynamic status (see Supplementary material online, *Figure S1*). Hypotension nevertheless progressed, requiring escalation of vasopressor support and re-initiation of VA-ECMO on Day 8 (see Supplementary material online, *Movie S3*). Despite a relatively low C-reactive protein level (1.13 mg/dL), serum IL-6 measured at the time of deterioration was markedly elevated (*Table 1*).

Given persistent haemodynamic instability despite maximal mechanical and pharmacologic support, emergent surgical resection of the left adrenal tumour was performed on Day 10. On gross examination, the tumour showed extensive haemorrhagic necrosis, consistent with a necrotic pheochromocytoma (*Figure 4B*). Histopathological examination confirmed pheochromocytoma. Despite tumour removal, severe circulatory failure persisted, requiring continued mechanical circulatory support. Progressive multiorgan failure subsequently developed, and the patient died on post-operative Day 2.

## Discussion

This case demonstrates how pheochromocytoma can present with a clinical phenotype nearly indistinguishable from fulminant myocarditis, resulting in substantial diagnostic uncertainty in the acute setting.

The initial working diagnosis of fulminant myocarditis was clinically appropriate.^4^ Fever, elevated inflammatory markers, severe global ventricular dysfunction, malignant ventricular arrhythmias, unobstructed coronary arteries, and cardiogenic shock collectively represent a classic fulminant myocarditis phenotype. Early endomyocardial biopsy and prompt initiation of immunomodulatory therapy were therefore consistent with contemporary management strategies.^4^ Transient biochemical and haemodynamic improvement following corticosteroid therapy reinforced the inflammatory myocarditis hypothesis and contributed to diagnostic anchoring. Although endomyocardial biopsy showed only minimal inflammatory infiltration, this finding did not exclude myocarditis because of potential sampling error and was interpreted in the context of the overall clinical presentation and transient clinical improvement. The diagnostic framework shifted after CT imaging revealed an adrenal mass. Importantly, sustained severe hypertension was absent throughout much of the clinical course. Although hypertensive crisis is classically associated with pheochromocytoma, some patients present predominantly with shock, arrhythmia, or acute heart failure.^1,2^ In such cases, reliance on persistent hypertension as a diagnostic clue may delay recognition of endocrine aetiologies. Because the patient was haemodynamically vulnerable after VA-ECMO withdrawal, vasodilation associated with α-blockade, potentially compounded by relative intravascular volume depletion, may have contributed to the subsequent deterioration; however, its relative contribution to the recurrent collapse cannot be determined.

Although a few reports have described pheochromocytoma presenting as fulminant myocarditis–like cardiogenic shock,^2,3^ such cases are often attributable to severe catecholamine-induced cardiomyopathy without clear inflammatory involvement. The educational value of the present case lies in the substantial diagnostic overlap between pheochromocytoma and fulminant myocarditis and the challenge of reassessing an initially plausible working diagnosis during rapidly evolving critical illness.

A central feature of this case was the magnitude of IL-6 elevation. In fulminant myocarditis, serum IL-6 levels are typically within the tens of pg/mL range,^5,6^ whereas our patient exhibited markedly higher levels despite corticosteroid therapy. Although limited by a single measurement after VA-ECMO re-initiation, the elevation appeared disproportionate to reported myocarditis cohorts. Its source could not be determined, and the finding remains hypothesis-generating rather than evidence of tumour-derived cytokine production.

Reports of IL-6–associated pheochromocytoma are scarce^7^ and have primarily described systemic inflammatory syndromes rather than cardiogenic shock. In this case, marked IL-6 elevation coexisted with catecholamine excess and severe myocardial dysfunction, raising the possibility that inflammatory activation accompanied the adrenergic cardiomyopathy; however, a causal contribution of IL-6 to myocardial dysfunction cannot be established. Catecholamine excess induces myocardial injury through β-adrenergic overstimulation and oxidative stress,^8^ whereas cytokine activation may further depress myocardial function via inflammatory signalling pathways.^9^

## Conclusions

Pheochromocytoma can mimic fulminant myocarditis in the presence of prominent inflammatory features. Marked IL-6 elevation may reflect concomitant inflammatory activation, although its source and mechanistic significance remain uncertain. Recognition of this pattern is important to avoid diagnostic anchoring and guide appropriate management, as even functional tumours can precipitate life-threatening cardiovascular complications.

## Lead author biography


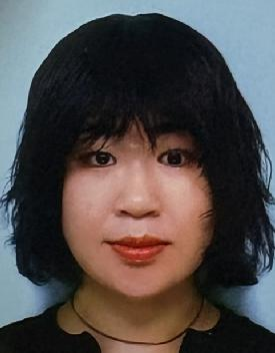
Dr Yuka Tanizaki graduated from Showa Medical University in 2017 and completed her clinical training at Showa Medical University Fujigaoka Hospital. She joined the Department of Cardiology in 2019 and obtained board certification as a cardiologist in 2024. Her clinical practice includes interventional cardiology and critical care medicine.

## Supplementary Material

ytag798_Supplementary_Data

## Data Availability

All data underlying this article are included within the article. No additional data are available.
