# Supplementary material for: Pheochromocytoma masquerading as fulminant myocarditis with marked interleukin-6 elevation: a case report
Source: Eur Heart J Case Rep. 2026 Oct 26;10(11):ytag798. doi: 10.1093/ehjcr/ytag798 (PMC13738081; doi:10.1093/ehjcr/ytag798)

Supplementary Table

Day	0	2	4	6	8	9	11
VA-ECMO flow (L/min)	3	3	2	Withdrawal	3	3	3.5
Impella support level	1	3	6	7	3	4	2
CHDF	−	−	−	−	+	+	+
Dobutamine (μg/kg/min)	0	0	0	0	0	0	0
Noradrenaline (μg/kg/min)	0	0	0	0	0	0	0.07
Vasopressin (U/h)	0	0	0	0	2	4	6
mPAP (mmHg)	23	31	37	18		7	
Laboratory data							
WBC (/μL)	16440	17390	28290	25250	32200	9590	350
Hb (g/dL)	16.1	10.8	10.2	7.8	8	9.7	7.6
Plt ($\times 10^3$/μL)	358	93	97	108	24	41	60
AST (U/L)	926	1095	519	195	233	1699	3665
ALT (U/L)	1054	979	973	533	201	1100	1189
Cr (mg/dL)	1.89	0.69	0.62	0.59	1.84	1.03	1.3
PT-INR	1.94	1.39	1.03	0.96	1.5	1.63	1.19
Lactate (mmol/L)	7.42	2.56	2.67	1.42	5.38	20.26	10.52
BNP (pg/mL)	63.2		1435.1			3944.3	

Supplementary Figure

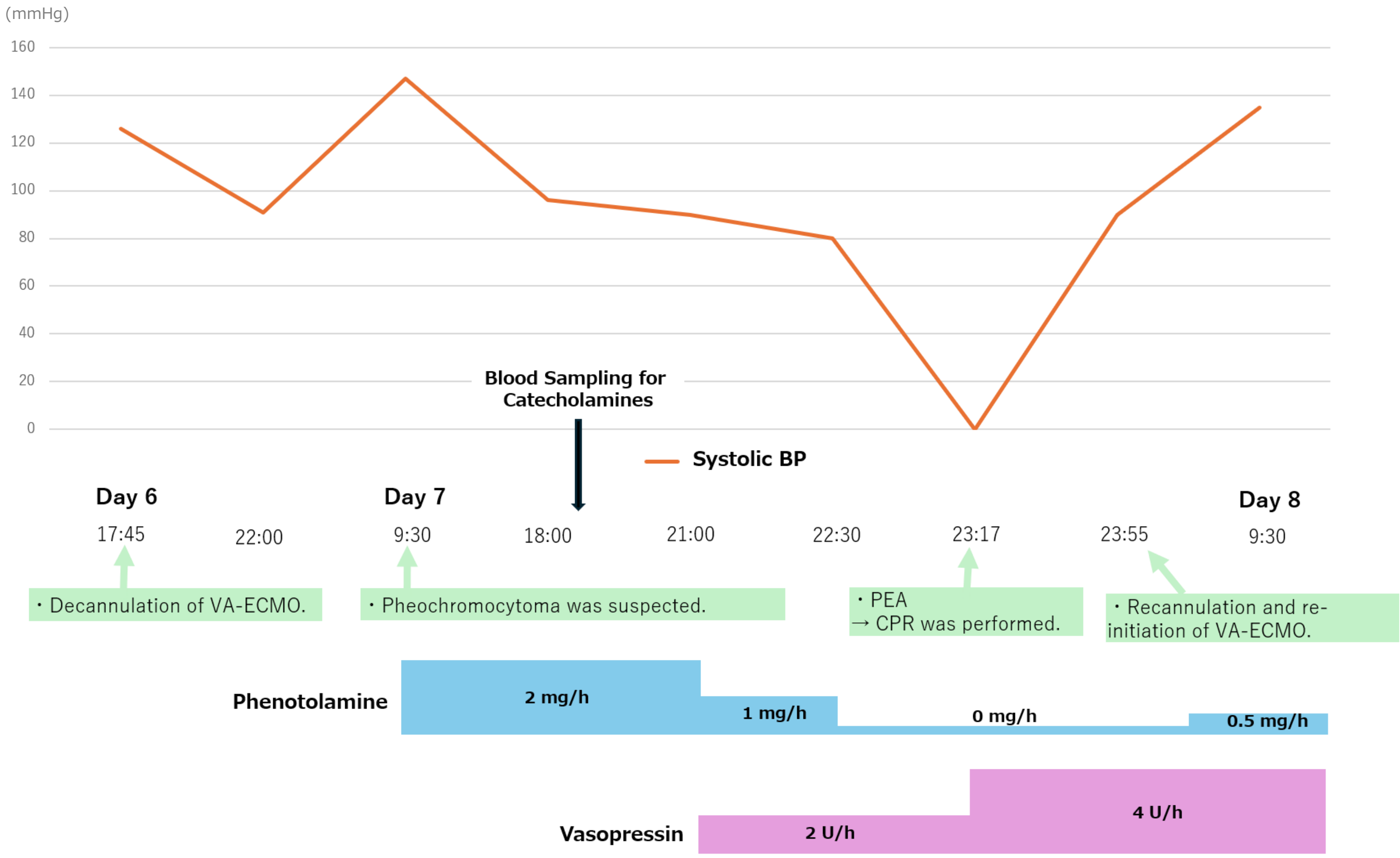

Supplement: Supplementary file 1 [file ytag798_supplementary_data.zip › 780_supp_fig.pdf]
