# Supplementary material for: Pheochromocytoma masquerading as fulminant myocarditis with marked interleukin-6 elevation: a case report
Source: Eur Heart J Case Rep. 2026 Oct 26;10(11):ytag798. doi: 10.1093/ehjcr/ytag798 (PMC13738081; doi:10.1093/ehjcr/ytag798)
Supplement: Supplementary file 1 [file ytag798_supplementary_data.zip › Supplementary_Legends.docx]

**Supplementary File Legends**

**Supplementary Movies**

Movie 1, at presentation; Movie 2, before VA-ECMO withdrawal; Movie 3, during recurrent hemodynamic collapse.

**Supplementary Table. Serial hemodynamic, mechanical circulatory support, vasoactive drug, and laboratory data during the clinical course**

ALT, alanine aminotransferase; AST, aspartate aminotransferase; BNP, B-type natriuretic peptide; CHDF, continuous hemodiafiltration; Cr, creatinine; Hb, hemoglobin; mPAP, mean pulmonary artery pressure; Plt, platelet count; PT-INR, prothrombin time–international normalized ratio; VA-ECMO, venoarterial extracorporeal membrane oxygenation; WBC, white blood cell count.

**Supplementary Figure. Hemodynamic course before and after initiation of phentolamine**
Time course of systolic blood pressure, phentolamine and vasopressin doses, and major clinical events from VA-ECMO withdrawal to recurrent haemodynamic collapse and VA-ECMO re-initiation. Blood sampling for catecholamine measurement was performed while exogenous noradrenaline and dobutamine were not being administered. CPR, cardiopulmonary resuscitation; PEA, pulseless electrical activity; VA-ECMO, venoarterial extracorporeal membrane oxygenation.
